# Oral *Astragalus* Root Supplementation for Mild to Moderate Chronic Kidney Disease: A Self-Controlled Case-Series

**DOI:** 10.3389/fphar.2022.775798

**Published:** 2022-03-01

**Authors:** Tetsuhiro Yoshino, Yuko Horiba, Masaru Mimura, Kenji Watanabe

**Affiliations:** ^1^ Center for Kampo Medicine, Keio University School of Medicine, Tokyo, Japan; ^2^ Department of Neuropsychiatry, Keio University School of Medicine, Tokyo, Japan

**Keywords:** chronic kidney disease, glomerular filtration rate (eGFR), astragalus root (Astragali radix, huangqi), kampo medicines (traditional Japanese medicine), renoprotective effect

## Abstract

In this self-controlled case series, we aimed to investigate the variation in estimated glomerular filtration rate (eGFR) after taking astragalus-containing preparations in patients with mild to moderate chronic kidney disease (CKD) by retrospectively reviewing their charts in our clinic. We set the inclusion criteria as first-visit patients aged 20 years or older presenting to our clinic between 1 October 2014, and 31 June 2019, and who were prescribed astragalus-containing herbal preparations for any reason. We calculated the mean eGFR from the readings taken 6 months before (pre) and after (post) the intake of astragalus-containing preparations for each participant. Among the 37 patients included in our final analysis, we found a statistically significant improvement in the eGFR after prescribing astragalus-containing preparations (pre, 66 ± 12 ml/min/1.73 m^2^ vs. post, 70 ± 14 ml/min/1.73 m^2^; *p* < 0.001 by paired *t*-test). Our results were consistent regardless of age, sex, CKD stage of the participants (G2 or G3), daily dosage of astragalus root, or duration of astragalus-containing preparations. No severe adverse reactions were recorded in the charts of the study participants. Our results suggest that there is eGFR improvement after taking astragalus-containing preparations in mild to moderate CKD cases as reported previously. The findings should be considered with caution due to major limitations such as small sample size without optimum control, short follow-up period, and incomplete data. Further adequately powered and designed studies are needed to confirm the efficacy and safety of the long-term use of astragalus root in patients with mild to moderate CKD.

## Introduction

Chronic kidney disease (CKD) is a long-term and globally widespread health condition. Moreover, this condition causes a huge economic burden due to end-stage renal disease requiring dialysis (Wang et al., 2016; Neuen et al., 2017; Luyckx et al., 2018).

Causes of CKD include high blood pressure, diabetes, glomerulonephritis, polycystic kidney disease, and long-term use of certain medications, such as non-steroidal anti-inflammatory drugs (Kazancioğlu, 2013). Unfortunately, there is no curative treatment for CKD itself. In addition to the current treatment, which focuses on delaying the disease by inhibiting the renin-angiotensin-aldosterone system, effective treatment options for CKD and its progression are required (Breyer and Susztak, 2016).

In Japan, astragalus root originates from the root of *Astragalus membranaceus* Bunge or *Astragalus mongolicus* Bunge (Leguminosae) (Motomura et al., 2009; Bi et al., 2020), which is taken orally as a powder or water extract. A previous meta-analysis of randomized control trials (RCTs) reported the efficacy of astragalus root on CKD based on estimated glomerular filtration rate (eGFR); serum creatinine, hemoglobin, and albumin levels; and proteinuria and albuminuria (Li et al., 2011; Zhang et al., 2014; Zhang et al., 2019). In addition, Chinese researchers have confirmed that astragalus injection is effective for hypertensive renal damage (Sun et al., 2012). These RCTs and observational studies mainly included patients with severe to end-stage CKD in stages G4 to G5 to avoid disease progression (Okuda et al., 2012). However, studies on the efficacy of astragalus root as an early intervention for patients with mild to moderate CKD in stages G2 to G3 are lacking.

In this study, we aimed to investigate the variation in estimated glomerular filtration rate (eGFR) after taking astragalus-containing preparations in patients with mild to moderate CKD by retrospectively reviewing their charts in our clinic. Our study focused on patients whose chief complaints were not CKD treatment, successfully including patients with mild to moderate CKD who took astragalus root unintentionally.

## Methods

### Study Design

This self-controlled case series was conducted at Keio University Hospital in Tokyo, Japan. The study was approved by the Keio University School of Medicine Institutional Review Board (approval no. 20100144), and the protocol was registered at the UMIN Clinical Trials Registry (unique ID: UMIN000020478).

### Participants

We set the inclusion criteria as first-visit patients aged 20 years or older presenting to our clinic at Keio University Hospital between 1 October 2014, and 31 June 2019, and who were prescribed astragalus-containing herbal preparations (e.g., boiogito, hochuekkito, and ogikenchuto) for any reason. We checked the adherence of these patients by following their medical records with prescriptions each time. We also allowed the continuous prescription of anti-hypertensive drugs, including renin-angiotensin-aldosterone system inhibitors. The exclusion criteria included patients who had no blood test before and/or after 6 months from the prescription of astragalus-containing preparation, had a baseline eGFR >90 ml/min/1.73 m^2^, and had an acute kidney injury (AKI) episode during the observational period. Written informed consent was obtained from all the participants prior to the study.

### Statistical Analysis

All statistical calculations and analyses were performed using the R software (version 4.1.1, 2021-08-10) with Rstudio (version 1.4.1717 2021-05-24 for macOS). Descriptive statistics were used to assess demographic characteristics. The eGFR values were extracted from the chart; however, they contained completely random timing observations on eGFR. Therefore, we calculated the mean eGFR value from the readings taken 6 months before (pre) and after (post) the intake of astragalus-containing preparations for each participant to compare the eGFR values between these two periods. Additionally, we compared the baseline eGFR value with the first observation before taking the astragalus-containing preparations or the last observation after taking the astragalus-containing preparations. We employed a paired *t*-test for these comparisons, and statistical significance was set at *p* < 0.05.

## Results

**FIGURE 1:**
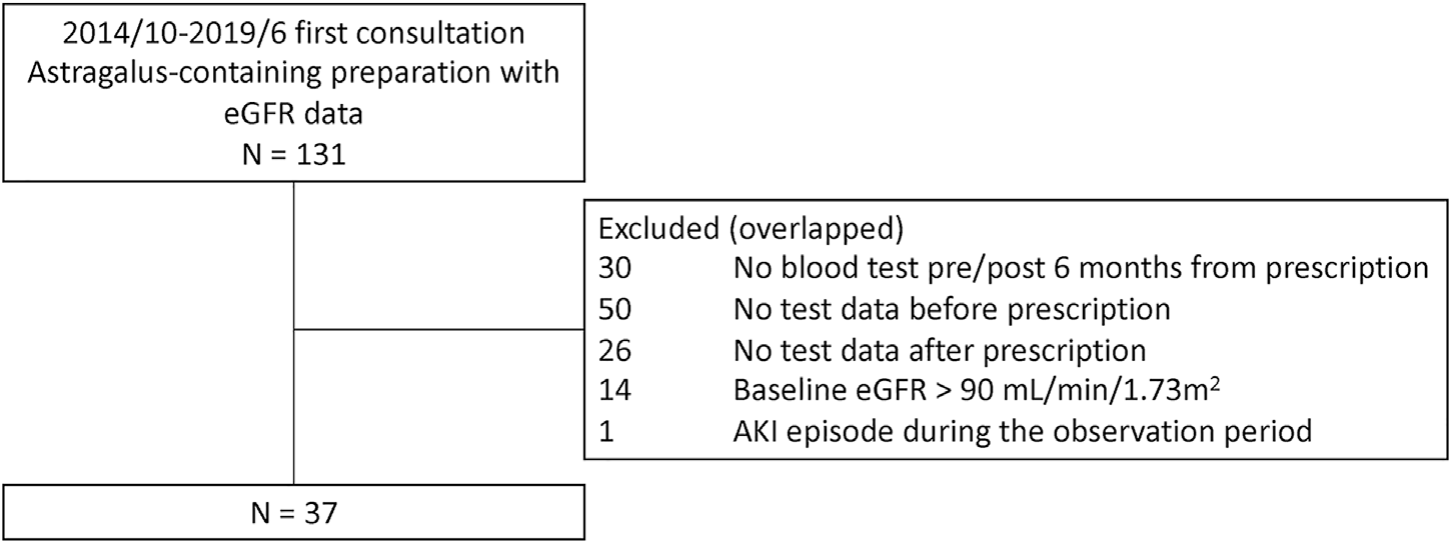
Patient exclusion flowchart. A total of 131 potential study participants were identified at the Keio University Hospital Kampo Clinic between 1 October 2014, and 31 June 2019, of which 37 patients were included in the final analysis.

A total of 131 potential study participants were identified (Figure 1). Of these, 94 patients were excluded due to insufficient eGFR data, mainly due to the lack of pre-treatment value in patients who had been referred to us from another hospital; 14 patients were excluded due to having a baseline eGFR >90 ml/min/1.73 m^2^; and one patient was excluded due to an AKI episode during the observation period. Thus, 37 patients were included in the final analysis (Figure 1; Table 1). The median dosage of astragalus root was 3.0 g (interquartile range; IQR, 3.0–4.0), and the median duration of astragalus-containing preparations was 3.4 months (IQR, 2.4–5.2).

**TABLE 1:** Background of participants.

	*N* = 37
Median age (yr)	—
Mean	61 ± 14
Median (IQR)	63.5 (50–73)
Female sex—no. (%)	28 (75)
Body mass index (kg/m^2^)	—
Mean	21.8 ± 4.1
Median (IQR)	21.5 (18.7–23.3)
Systolic blood pressure (mmHg)	—
Mean	124 ± 20
Median (IQR)	124 (113–137)
Diastolic blood pressure (mmHg)	—
Mean	74 ± 14
Median (IQR)	73 (66–77)
Hemoglobin (mg/dl)	—
Mean	12.7 ± 1.5
Median (IQR)	13.0 (11.9–13.6)
Missing data	1 (2.7)
Albumin (g/dl)	—
Mean	4.2 ± 0.3
Median (IQR)	4.1 (4.0–4.4)
Missing data	5 (13.5)
Estimated glomerular filtration rate (eGFR)	—
Mean—ml/min/1.73 m^2^	66 ± 13
Median (min, IQR, max)	67 (34, 59-74, 88)
G2 (60≦eGFR<90)	28 (75.7)
G3 (30≦eGFR<60)	9 (24.3)
Distribution of Urinary protein—no. (%)	—
<30	14 (37.8)
30 to <300	3 (8.1)
≧300	1 (2.7)
Missing data	19 (51.4)
Comorbidities (only in two or more participants)—no. (%)	
Breast cancer	4 (10.8)
Systemic lupus erythematosus	3 (8.1)
Sjogren Syndrome	3 (8.1)
Lung cancer	3 (8.1)
Prostate cancer	2 (5.4)
Urethral cancer	2 (5.4)
Lumbar canal stenosis	2 (5.4)
Baseline medications—no. (%)	
Renin-angiotensin system inhibitor	7 (18.9)
Diuretic	4 (10.8)
Statin	5 (13.5)
Glucose-lowering therapy	3 (8.1)
*Astragalus* root daily dosage (Gram)	—
Mean	3.2 ± 0.98
Median (IQR)	3.0 (3.0–4.0)
Distribution—no. (%)	—
5.0	3 (8.1)
4.0	12 (32.4)
3.0	13 (35.1)
2.7	2 (5.4)
2.0	1 (2.7)
1.8	3 (8.1)
1.5	3 (8.1)
Duration of astragalus-containing preparations (months)	
Mean	3.5 ± 1.8
Median (IQR)	3.4 (2.4–5.2)

IQR, interquartile range; CKD, chronic kidney disease; Plus-minus values are means ± standard deviation.

**FIGURE 2:**
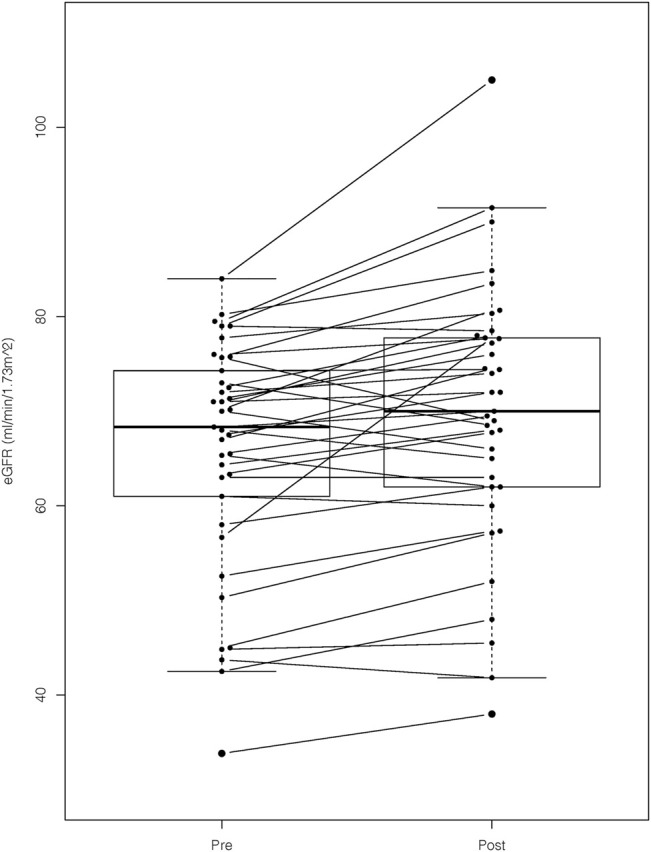
The eGFR values before and after prescription of astragalus-containing preparations. We confirmed a statistically significant improvement of estimated glomerular filtration rate (eGFR) after prescription of astragalus-containing preparations. The mean eGFR value of the pre- and post-astaragalus prescription readings were determined by obtaining the mean value of all the available values 6 months before and after administering astragalus-containing preparations. We applied the paired *t*-test for this comparison (*p-*value < 0.001).

We confirmed a statistically significant improvement in the mean eGFR after prescription of astragalus-containing preparations (pre, 66 ± 12 ml/min/1.73 m^2^ vs. post, 70 ± 14 ml/min/1.73 m2; p < 0.001; Figure 2). The comparison between the baseline eGFR value and the first observation before taking the astragalus-containing preparations showed no statistically significant differences (first, 66 ± 13 ml/min/1.73 m^2^ vs. baseline, 66 ± 13 ml/min/1.73 m^2^; *p* = 0.63). Contrarily, the comparison between the baseline eGFR value and the last observation after taking the astragalus-containing preparations showed a statistically significant difference (baseline, 66 ± 13 ml/min/1.73 m^2^ vs. the last, 71 ± 14 ml/min/1.73 m^2^; *p* < 0.001). Plasma concentration of hemoglobin or albumin did not differ significantly, and we hesitated to compare the degree of proteinuria due to lacking urinalysis data. Our results were consistent regardless of age, sex, CKD stage of the participants (G2 or G3), daily dosage of astragalus root, or duration of astragalus-containing preparations.

Furthermore, we performed subgroup analysis by dividing the participants based on the presence of eGFR improvement in the observational period, but we did not find any difference in age, sex, CKD stage, daily dosage of astragalus root, or duration of astragalus-containing preparations.

There was no severe adverse reaction recorded in the study participants’ charts.

## Discussion

Our results suggest that there is improvement in the eGFR after taking astragalus-containing preparations for mild to moderate CKD. Our study successfully included patients with mild to moderate CKD who unintentionally took astragalus root.

Previous studies have reported on the various bioactive chemicals in astragalus root (Bratkov et al., 2016) and related pharmacological pathways involving molecular targets, including antioxidants, anti-inflammatory response, and antiapoptosis, and cell cycle regulation (Zhao et al., 2021). *Astragalus* root is also effective in reducing fasting blood glucose and albuminuria levels, in reversing the glomerular hyperfiltration state, and in ameliorating the pathological changes of early diabetic nephropathy in animal models (Zhang et al., 2009). Notably, one of the potential mechanisms of the renoprotective effect of astragalus root extract is its natriuretic properties (Ai et al., 2008). Despite these findings, the possibility of a renoprotective effect in astragalus root is still up to discussion. Several studies have shown the muscle protective effect of astragalus polysaccharide (Zheng et al., 2020) that prevents creatin release.

This study had several limitations. First, the CKD-G stages of our participants were G2 and G3, and it is unclear whether our results can be applied to patients with CKD stage G4 or G5. Although, previous studies have already reported favorable effects of astragalus roots in this population. Second, previous studies employed a higher daily dosage of *Astragalus membranaceus*, possibly suggesting higher efficacy if a higher dosage was used in our study population. Third, our study was observational, including small sample size, obtaining incomplete renal data, and the daily dosage and dosing duration varied significantly. Fourth, we could not conclude the efficacy and safety of long-term interventions in our short follow-up study. Fifth, we did not compare patients treated with Kampo formulas without astragalus root with the present self-controlled study. Therefore, we additionally performed regression analysis and confirmed the negative eGFR trend over time before astragalus-containing preparations (Table 2). Lastly, we excluded one patient who experienced a better course after taking astragalus-containing preparations due to having an AKI during the observational period.

**TABLE 2:** Effects of predictor variables.

Estimates (95% CI)	Pre		Post	
	(Intercept)	month	(Intercept)	month
Fixed-effect model (without random effect)	58 (54–63)	−0.84 (−2.1–0.44)	60 (57–63)	2.3 (1.0–3.5)
Mixed-effects model (with random effect)	58 (56–61)	−2.0 (−3.4–0.56)	61 (59–64)	2.2 (0.55–3.9)

a We employed a mixed-effects model aside from a fixed-effect model because the mixed-effects model is flexible enough to account for different numbers of observations per subject. Mixed-effects linear regression analysis was based on the continuous dependent variable eGFR, value and included one random effect for slope and two fixed effects for intercept and month.

In conclusion, Our results suggest that there is eGFR improvement after taking astragalus-containing preparations in mild to moderate CKD cases as reported previously. The findings should be considered with caution due to major limitations such as small sample size without optimum control, short follow-up period, and incomplete data. Further adequately powered and designed studies are needed to confirm the efficacy and safety of the long-term use of astragalus root in patients with mild to moderate CKD.

## Data Availability

The data that support the findings of this study are not publicly available due to their containing information that could compromise the privacy of research participants but are available from the corresponding author TY upon reasonable request.
